# A Proposal to Develop New References for Serum IGF-I Levels in Children

**DOI:** 10.4274/jcrpe.galenos.2020.2020.0040

**Published:** 2020-06-03

**Authors:** Michael B. Ranke

**Affiliations:** 1 University Children’s Hospital, Clinic of Pediatric Endocrinology, Tübingen, Germany

***Commentary to Wit et al. 2019 “A proposal for the interpretation of the serum IGF-I concentration as part of laboratory screening in children with growth failure” J Clin Res Pediatr Endocrinol. 2019 Dec 17. ***

***doi: 10.4274/jcrpe.galenos.2019.2019.0176***

## 

Wit and collaborators (1) have again approached the issue of the value of IGF-I serum levels as a tool for diagnosing growth hormone deficiency (GHD). They have thoughtfully reviewed the existing literature and deliberated that the cut-off in terms of standard deviation (SD) score (SDS) should be interpreted based on the pre-test likelihood of GHD. I recommend reading this interesting article. In short, the authors concluded: In the case the pre-test likelihood for GHD - based on anamnestic information, physical findings, anthropometric measurements and laboratory results not specifically related to GHD - is high (>50%) further testing (including measurements of GH secretion) is recommended even if IGF-I levels are in the normal range. However, if the pre-test likelihood is low (<10%) only very low IGF-I levels (<-2 SDS) qualify these children for a further diagnostic work-up.

The diagnosis of GHD in childhood is in fact complex and is subject to an ongoing, partly controversial discussion (2). A number of - often rather less well-defined terms - have been used to subclassify GHD, including congenital, acquired, idiopathic, with or without (isolated) other pituitary hormone deficits, severe, less severe, total, and partial GHD (3). In addition, the clinical picture, as well as the diagnostic tools and criteria applied, are dependent on the age of the patient. The key issue remains the methodologically difficult quantification of GH secretion, which means defining the individual GH secretion as being insufficient (deficient) compared to normal. Components of the IGF system [IGF-I, IGF-binding protein-3 (IGFBP-3), and others] are qualitatively dependent on GH secretion. Their serum levels (IGF-1, IGFBP-3) have been proven to be positively correlated with the spontaneously secreted amount of GH in children and adolescents (4). Thus the attempt to use their blood levels (and their standardized derivatives) as a potential diagnostic indicator of GHD (5,6,7) is rational.

Modern classification system which are placing IGF-I into the center of a classification even distinguish between disorders with primary IGF-deficiency (such as the inability to primarily produce IGF-1) from those with secondary IGF- deficiency (such as GH deficiency) (3,8). Serum levels of IGF-I (or IGFBP-3) show little circadian variance (9) and their measurement is well standardized and generally accessible (10), factors which recommend their use as a diagnostic step before specific GH testing.

The idea proposed by Wit et al (1) to establish a pre-test likelihood of GHD makes sense and is probably intuitively used by every physician diagnosing short children. To transform this into an empirically based scoring system is certainly difficult, but potentially doable. However, in my view, much of the problem of using IGF-I (IGFBP-3) as diagnostic tools is caused by the available references. Since the measurement of Somatomedin C = IGF-I had become available by means of radioimmunoassay (11) several authors have published references for age and sex based on children, adolescents or adults from large cohorts using various immunoassay techniques (6,12,13,14,15,16). In general, these references show the following qualitative characteristics for IGF-I: 1. During the childhood years serum levels show a steady increase from very low levels at birth onwards with no quantitative differences between the sexes. 2. During puberty there is a steep increment with a peak at about mid-puberty with levels in females exceeding those in males. 3. Thereafter there is a gradual decline - females remaining higher than males - reaching very low levels during senescence. The levels in all age groups are not distributed by a Gaussian characteristic but are skewed positively (to the right of the mean). The latter phenomenon is observed to a lesser degree for IGFBP-3 as compared to IGF-I. In order to express the relative magnitude of the difference of a patient’s value from the reference mean we are used to calculating the SDS for age: SDS = (patient’s value - mean of patient’s age-related reference) divided by the SD of mean of patient’s age-related reference. If parameters are skewed specific mathematical transformations are needed to calculate means and SDs for a given age as the basis for such a calculation. In order to approximate towards a normal distribution of the references several methods have been applied such as logarithmic transformation or square root transformation (6,13,15,16).

The author always found it remarkable that that quantitative spread of the normal range for serum IGF-I (and other IGF parameters) for a given age is relatively high compared to other biological parameters. For example, in a 7 year old child, the normal range of serum IGF-I levels is from about 60 to about 250 µg/L, while for standing height it is only from about 114 to 134 centimeters. Authors who have reported references of IGF parameters have shown that IGF-I levels in serum of children are not only dependent on age and sex but also on pubertal stage and body mass index (BMI). Therefore it appears to be very likely that the enormously wide range (for the usual denominators age and sex) of normal of IGF-I levels is indeed caused by the variability of other effectors, which are not accounted for. The quantitative impact of pubertal development as expressed in terms of the rather crude Tanner stages has been investigated rather extensively (12,16). However, for the diagnosis of idiopathic GHD this appears to be of little practical relevance since the children in question have a delayed development and normal references for children in pubertal age but without puberty would be needed.

Alberti and collaborators (16) calculated that in short children a change of one SDS of height - or of BMI - corresponds to +/- 0.2 SDS in IGF-I for age and sex. Thus, if a short and obese boy (e.g. BMI = +2.5 SDS], has an IGF-I level for age of – 0.5 SDS, this figure must be interpreted as being about 0.5 SDS too low for the condition of overweight. In addition, some authors have observed that height and weight also play an independent role for IGF-I levels. If such relevant co-variants (pubertal stage, body composition, and others) should play a major role for IGF serum levels they should also be measured when establishing references of normally tall children. The co-variants should also be truly quantified (e.g. levels of sex hormones rather than just rough pubertal staging; exact non-invasively measured masses of muscle, fat, bone, and tissue size rather than calculating BMI alone; - and using automatically determined bone age in addition to chronological age; etc.). 

If the quantitative effect of these factors on the reference values were known the expected mean reference value (and its SD) for the individual patient in question could be calculated with the help of a multivariate regression algorithm. A somewhat similar approach has been used for references of serum Leptin levels by Blum et al (17). Such individually calculated (predicted) reference figures (mean, SD) could then be used to calculate an individual (adjusted for the individual co-variants) SD-score. The approach of developing such “conditional“ references should at least be attempted for children during the (prepubertal) childhood age (about 4-11 years), at an age range when isolated, idiopathic GHD, which has the lowest pre-test likelihood, is diagnosed most frequently in childhood. The information collected for establishing the reference of Bereket et al (15) or Alberti et al (16) could probably be used for such an analysis. Such a novel approach may be an additional step to further substantiate the diagnostic value of measuring IGF-parameters in short children and reach a higher likelihood of GHD before further extensive testing. Multidimensional reference region combining the levels of various IGF parameters as used in adults (18) may perhaps also augment their diagnostic value in short children.
